# Highly efficient removal of ammonia nitrogen from wastewater by dielectrophoresis-enhanced adsorption

**DOI:** 10.7717/peerj.5001

**Published:** 2018-06-15

**Authors:** Dongyang Liu, Chenyang Cui, Yanhong Wu, Huiying Chen, Junfeng Geng, Jianxin Xia

**Affiliations:** 1 College of Life and Environmental Science, Minzu University of China, Beijing, China; 2 Institute for Materials Research and Innovation, Institute for Renewable Energy and Environmental Technologies, University of Bolton, Bolton, UK

**Keywords:** Dielectrophoresis, Ammonia nitrogen, Adsorption, Wastewater, Zeolite

## Abstract

A new approach, based on dielectrophoresis (DEP), was developed in this work to enhance traditional adsorption for the removal of ammonia nitrogen (NH_3_-N) from wastewater. The factors that affected the removal efficiency were systematically investigated, which allowed us to determine optimal operation parameters. With this new method we found that the removal efficiency was significantly improved from 66.7% by adsorption only to 95% by adsorption-DEP using titanium metal mesh as electrodes of the DEP and zeolite as the absorbent material. In addition, the dosage of the absorbent/zeolite and the processing time needed for the removal were greatly reduced after the introduction of DEP into the process. In addition, a very low discharge concentration (C, 1.5 mg/L) of NH_3_-N was achieved by the new method, which well met the discharge criterion of C < 8 mg/L (the emission standard of pollutants for rare earth industry in China).

## Introduction

Nitrogen is a basic structural element of biological proteins in life. In water, one of the major forms of nitrogen is ammonia nitrogen (NH_3_-N). NH_3_-N is essential to living organisms, but exceeding threshold level could cause environmental pollution (Rožić et al., 2000). A large amount of NH_3_-N could cause serious disorder of natural materials circulation, which threats human health. According to WHO guidelines for drinking-water quality, the concentration of NH_3_-N should not exceed 1.5 mg/L in drinking water (Gorchev & Ozolins, 1984). High level NH_3_-N present in water may cause eutrophication to occur (Blomqvist, Pettersson & Hyenstrand, 2008; Huang et al., 2014; Lin et al., 2009). Under this circumstance, NH_3_-N could be toxic to most cultured animals (Chang et al., 2015; Ebeling, Timmons & Bisogni, 2006; He et al., 2011). NH_3_-N may also be oxidized into nitrite, when this is taken into human body, could cause cancer (Kim et al., 2005; Li et al., 2009; Wang, Kmiya & Okuhara, 2007).

How to efficiently remove NH_3_-N from wastewater has, as a research topic, attracted great interests in recent years. The existing methods include ammonia stripping (Bonmatí & Flotats, 2003; Liao, Chen & Lo, 1995), chemical precipitation (Diwani et al., 2007; Li, Zhao & Hao, 1999; Ryu, Kim & Lee, 2008), electrochemical conversion (Kim et al., 2006) and membrane separation (Ali et al., 2010; Domingos et al., 2011). However, none of these can provide an ideal way for a large-scale operation for each of these methods has its own major shortcoming in terms of either the removal efficiency or the processing cost. Ammonia stripping is only suitable to high concentrations of NH_3_-N and the processing cost is high (Zhang, Lee & Jahng, 2011). Chemical precipitation would need high concentrations of NH_3_-N and additional reagents, which may cause secondary pollution (Diwani et al., 2007). Electrochemical method is typically of high cost for the method is energy consummative and needs precious metals as the electrode materials (Kim et al., 2006). Membrane separation could be efficient but it usually uses expensive materials to construct the membrane. Clearly, it is significant to develop a low-cost, high-efficiency method to remove NH_3_-N from wastewater.

On the other hand, adsorption technique has long been used to remove various types of impurities from wastewater due to its appreciable removal efficiency and relatively low cost (Zheng, Xie & Wang, 2012). Moreover it has been reported that zeolite shows a strong adsorption activity on NH_3_-N (Watanabe et al., 2003; Wan et al., 2017). It has thus been exploited to remove NH_3_-N from wastewater (Delkash, Bakhshayesh & Kazemian, 2015; Halim et al., 2009; Wang & Peng, 2010). However, the removal efficiency by adsorption itself with zeolite is not high enough, and the removal speed is quite slow (also see our experimental data in this paper), which makes us to consider how to significantly enhance the adsorption while still keep the operation at a relatively low cost. Here we report that with the support from dielectrophoresis (DEP), both the removal speed and removal efficiency can be largely improved. DEP is a powerful tool that can be used to manipulate polarized particles suspended in fluid media in a non-uniform applied electric field. In this work, DEP could have helped to effectively trap targeted zeolite particles (with NH_3_-N attached on top) onto the electrodes so that the post-process separating of the solid from wastewater and cleaning to the operation device become easy and handy.

## Materials and Methods

Ammonium chloride (Analytical reagent, China National Medicines Corporation Ltd, Beijing, China) was used as the source of NH_3_-N. Ultrapure water was used for preparation of the solutions. Adsorbents were bentonite (China National Medicines Corporation Ltd, Beijing, China) and zeolite (Tianjin Fu Chen Chemical Reagents Factory, Tianjin, China). Stainless steel wire mesh and titanium mesh were used as the electrodes to determine the optimal operation parameters for achieving maximum removal efficiency of NH_3_-N from water.

**Figure 1:**
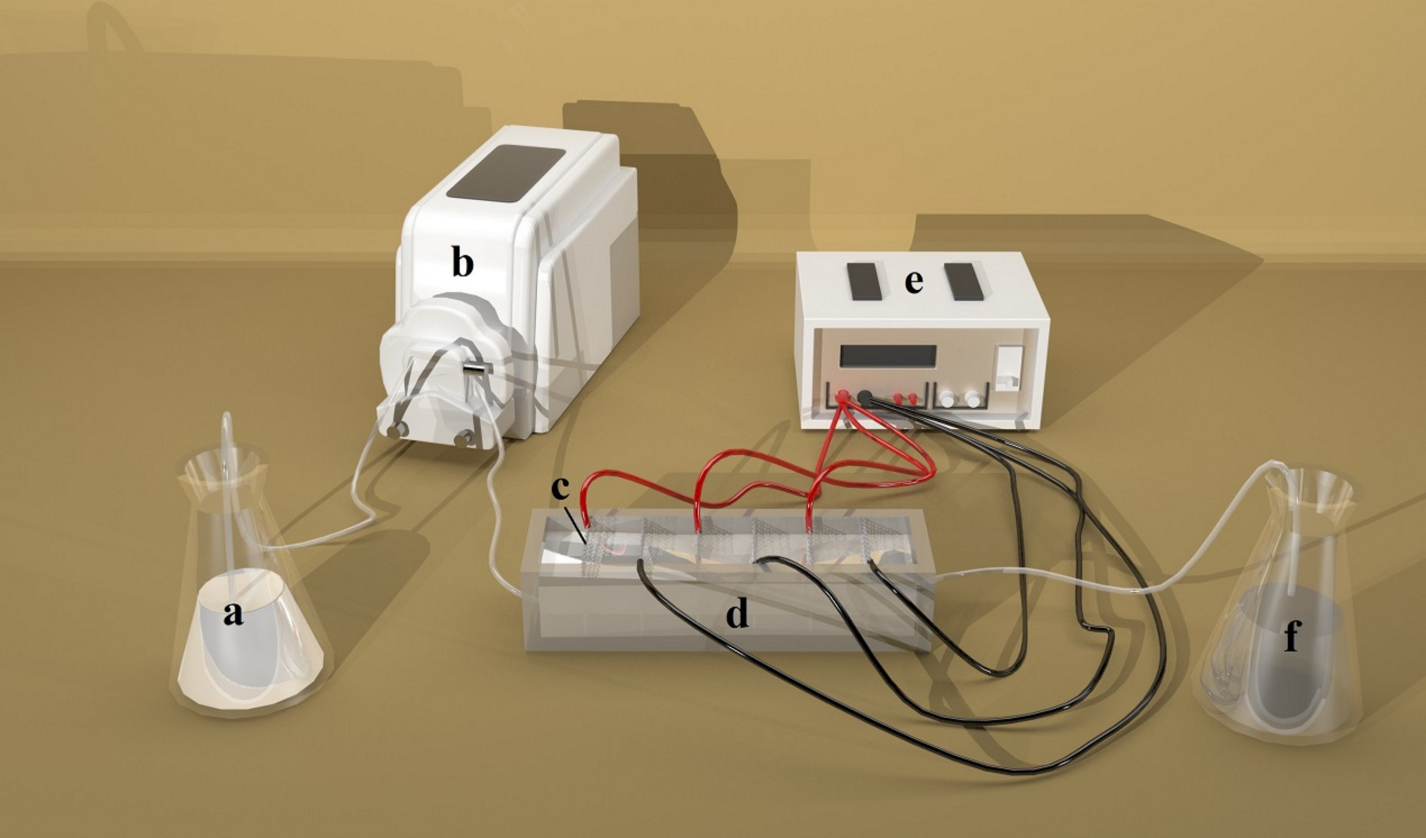
Schematic diagram of DEP apparatus. (A) Storage conical flasks; (B) Pump; (C) Titanium metal mesh; (D) Fluid vessel; (E) Direct current power source; (F). Reception conical flasks. Photo by Dongyang Liu.

The experimental apparatus is shown in Fig. 1. A direct current power device (PS-305DM; Longwei Instruments (HK) Co., Ltd, Hong Kong, China) was used as the power source. NH_3_-N was measured by NH_3_-N Analyzer (DWS-296; Shanghai Electronics Science Instrument Co., Ltd, Shanghai, China). Zeolite particles and the electrodes were characterized by scanning electron microscopy (SEM) (S-4800; Shimadzu, Kyoto, Japan).

Adsorption experiments were performed at room temperature (25 °C) in a series of conical flasks containing NH_3_-N solutions with initial concentration of 30 mg/L, except the experiment for effects of initial concentration. A total of 2 g/L adsorbent (except the experiment for effects of dosages) was first added to a solution and the suspension was then stirred for 20 min for adsorption to occur. This was followed up by a series of DEP processes carried out at room temperature (25 °C) with a home-made apparatus. Ten electrodes were installed to the slots on sidewalls of the vessel (Fig. 1). The distance within each pair of electrodes was set at 10 mm. A direct-current power device was used to supply voltage to the electrodes. Following a prior adsorption treatment, the suspension was forced by a pump to flow through the DEP device with the flow rate of 16 mL/min. The voltage was applied to mesh electrodes, which could create non-uniform electric field, and then, the zeolite particles with NH_3_-N attached on the top were trapped by the electrodes. The DEP processing time was 20 min, except the experiment for effects of processing time.

## Results

### Effects of processing factors on the removal efficiency

**Figure 2:**
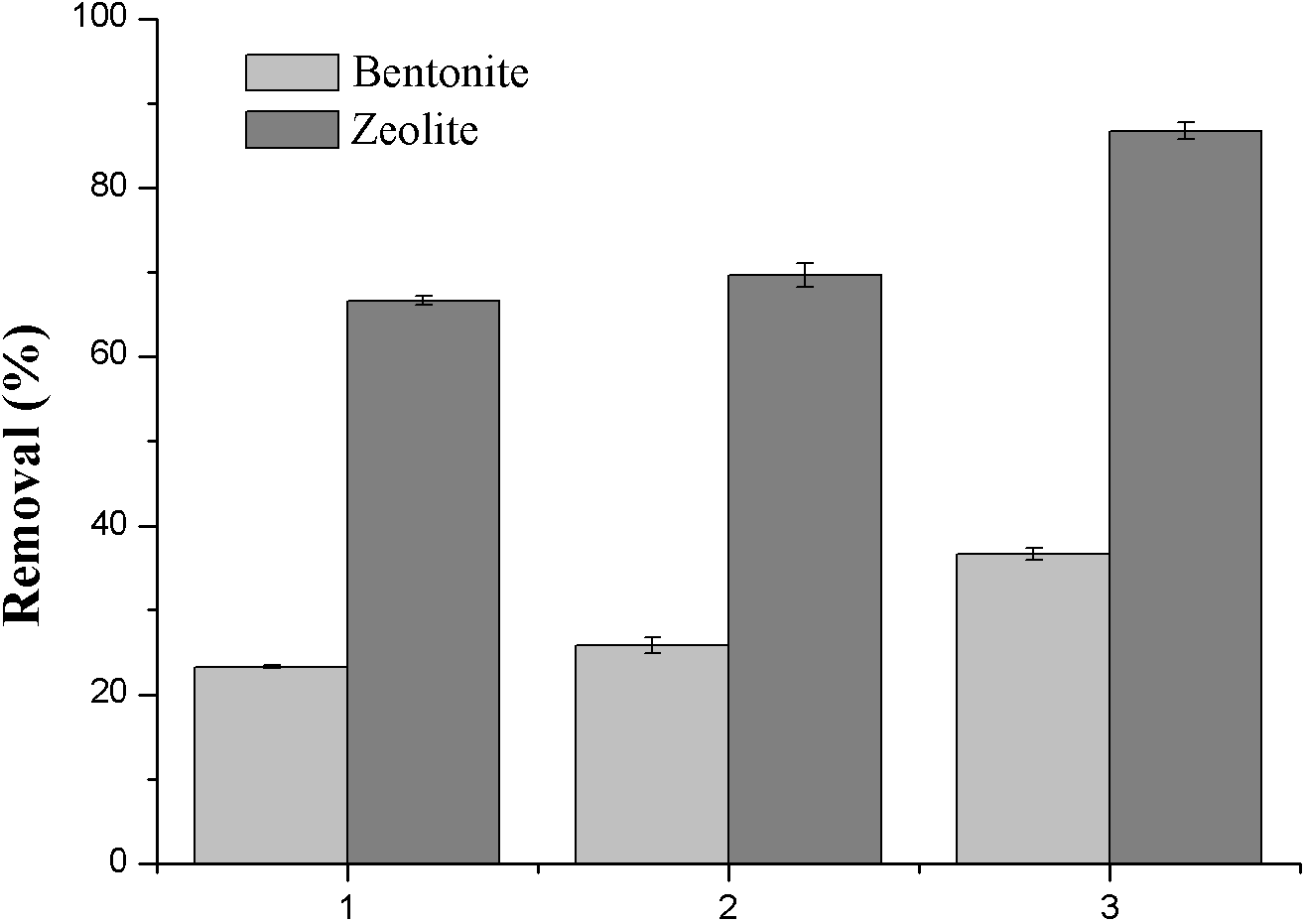
Effect of absorbents and electrode materials on the removal efficiency of NH_3_-N. (1) Using ADS only; (2) using ADS-DEP with stainless steel mesh as the electrodes; (3) using ADS-DEP with titanium mesh as the electrodes.

**Figure 3:**
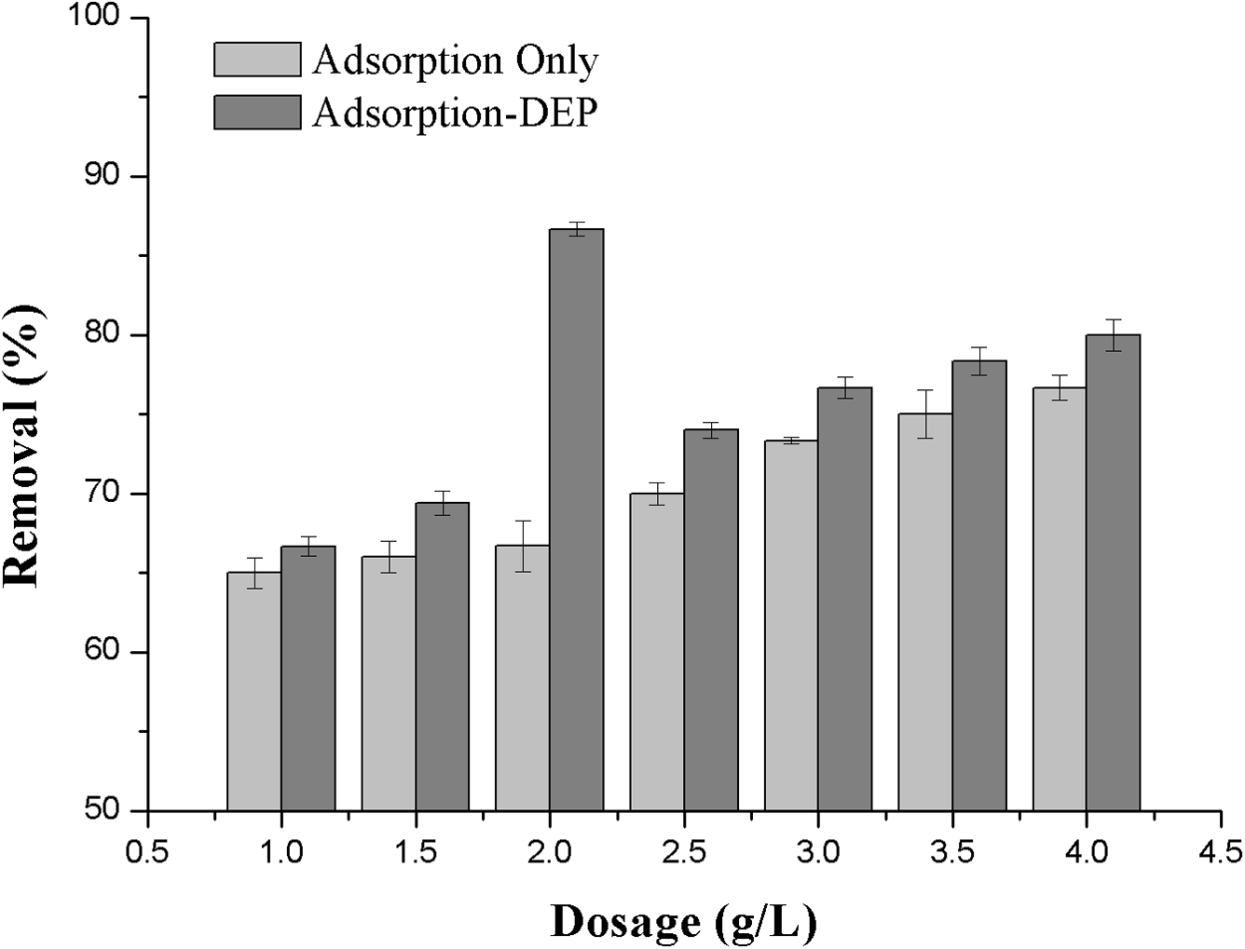
Effect of absorbent dosage on the removal efficiency of NH_3_-N.

**Figure 4:**
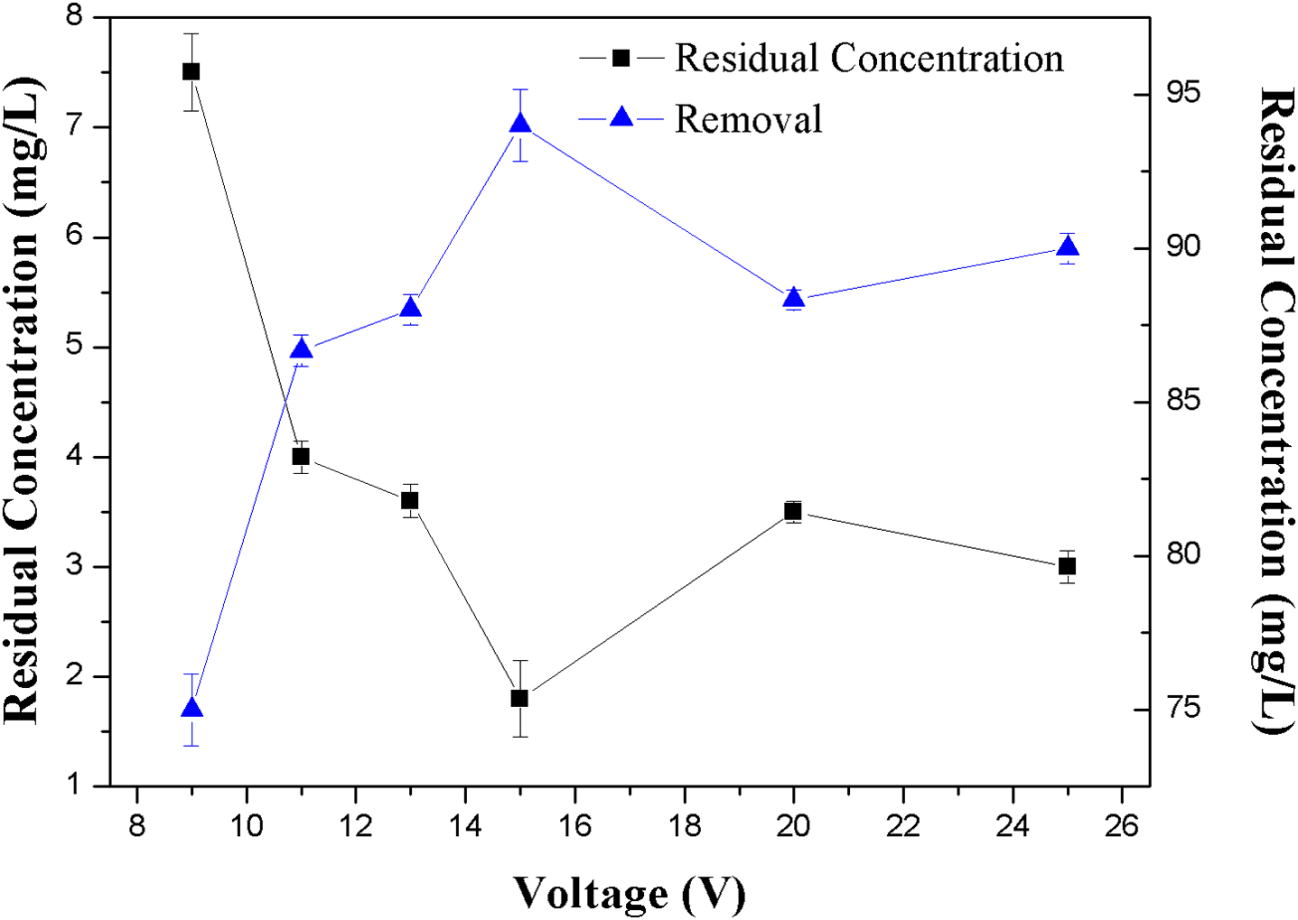
Effect of the voltage on the removal efficiency of NH_3_-N.

**Figure 5:**
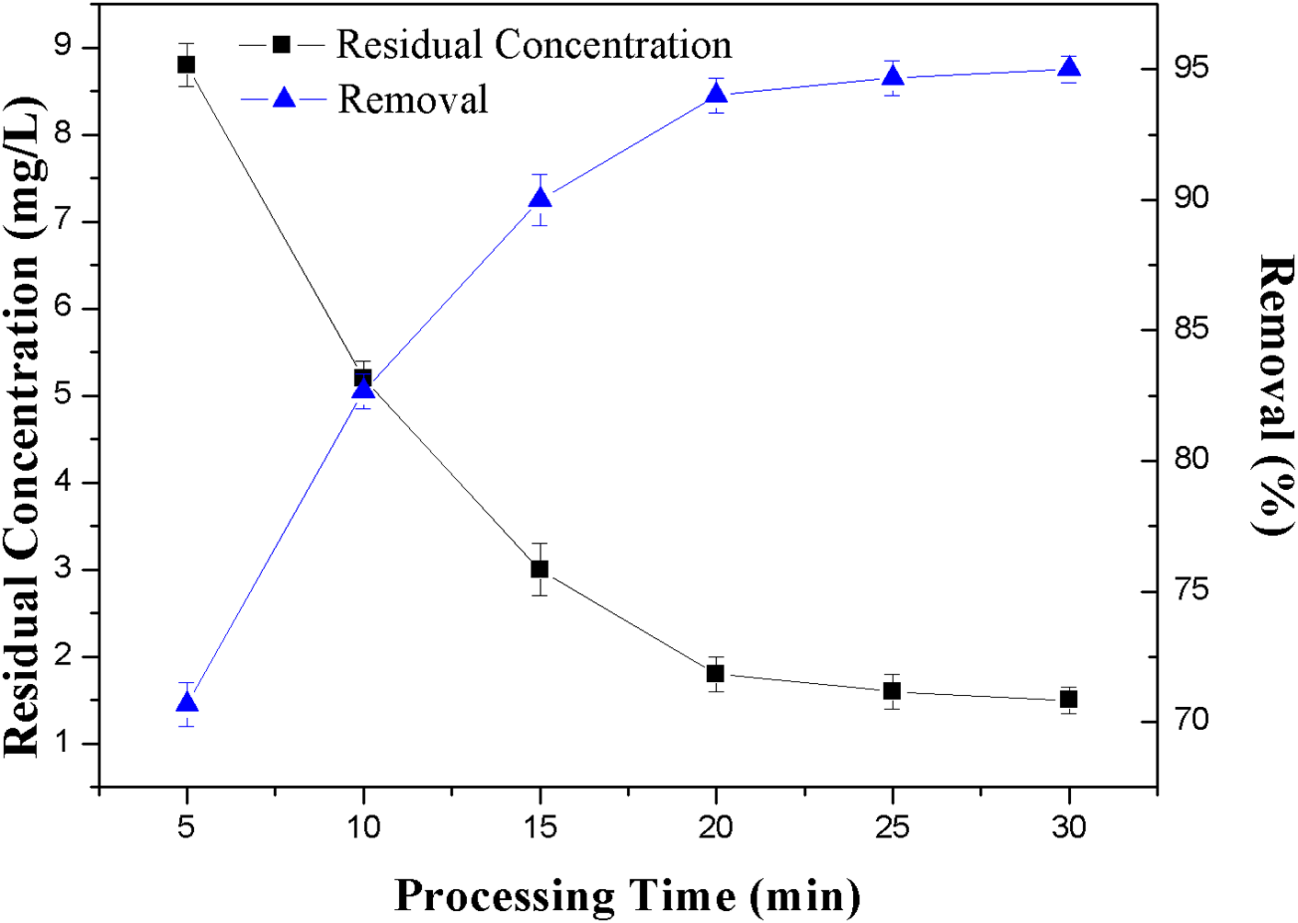
Effect of processing time on the removal rate of NH_3_-N.

**Figure 6:**
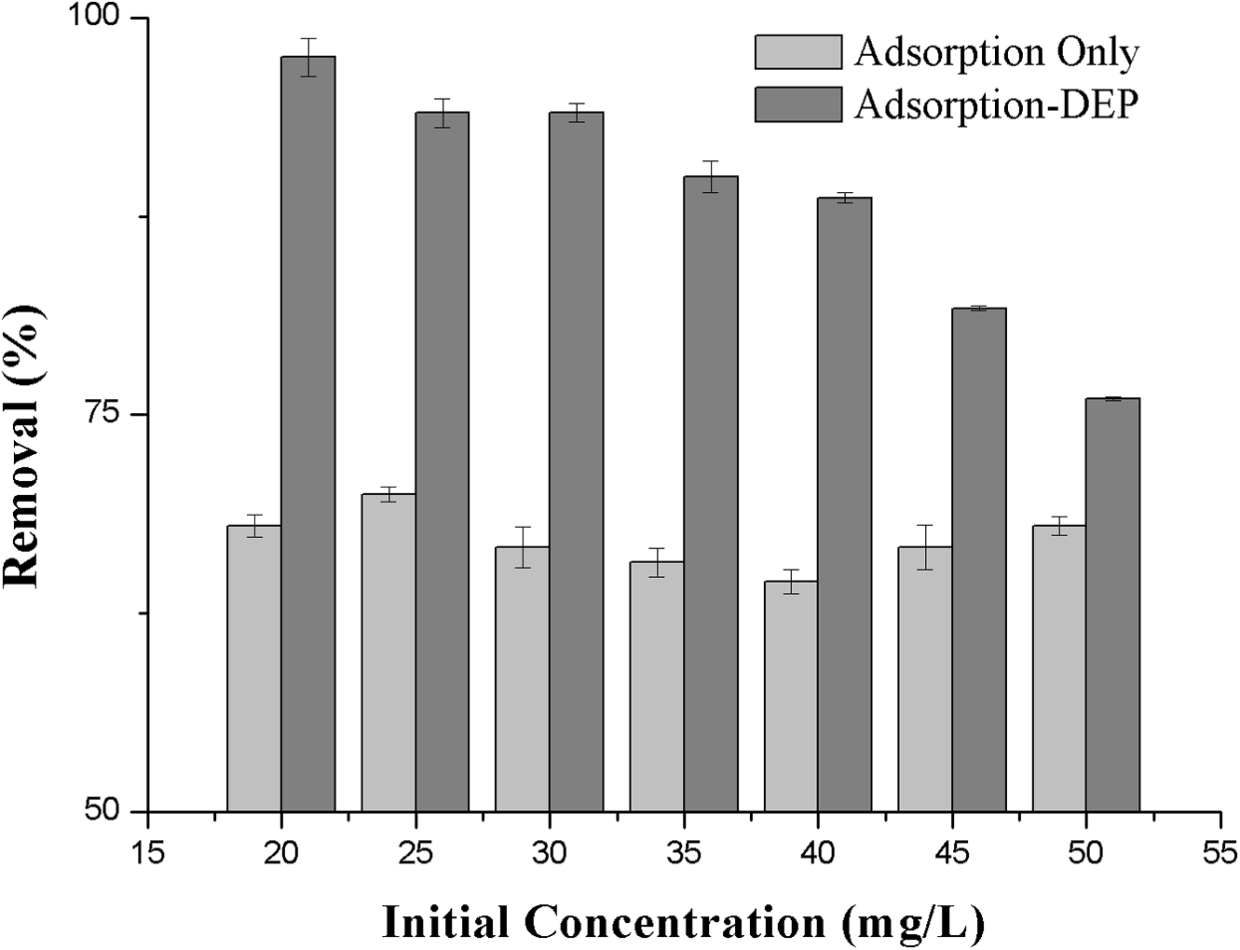
Effect of the initial concentration on the removal efficiency of NH_3_-N.

The factors of removal of NH_3_-N by DEP-enhanced adsorption are given in Figs. 2–6.

#### Effects of adsorbents and electrode materials

In order to select suitable materials acting as adsorbent for the adsorption and electrodes for DEP, initial tests were carried out. Figure 2 shows the variation of the removal efficiency by using 2 g/L zeolite and bentonite as the adsorbent separately and stainless steel mesh and titanium mesh as the electrodes, with initial NH_3_-N concentration of 30 mg/L and voltage of 11 V. The removal efficiency of NH_3_-N was only 23.3% using bentonite adsorption only, while this figure was much higher (66.7%) using zeolite (case 1 in Fig. 2). Moreover, both figures were slightly increased after the introduction of DEP using stainless steel mesh as the electrodes (case 2 in Fig. 2). However, it was observed that the removal efficiency was largely enhanced when titanium mesh electrodes were employed in the DEP, resulting in much better figures, i.e., 37% with bentonite and 87% with zeolite (case 3 in Fig. 2). Therefore, the titanium mesh electrodes and zeolite were used in the following experiments.

#### Effects of dosages for different processes

The zeolite dosage exerts a strong effect on the removal efficiency in both adsorption only and adsorption-DEP processes. The effect of zeolite dose, varying from 1 to 4 g/L, on removal of NH_3_-N is presented in Fig. 4. The initial NH_3_-N concentration was 30 mg/L with applied voltage of 11 V.

It can be seen from Fig. 3 that in an adsorption process only, the removal efficiency of NH_3_-N increased continuously with the increase of the zeolite dosage. In the adsorption-DEP process, it is interesting to see the experimental data that clearly showed the optimal dosage of zeolite (in this case, it was about 2.0 g/L), which corresponded to a peak removal efficiency of 86.7% (Fig. 3).

#### Effects of voltages

Apart from the dosage effect, the voltage applied to the electrodes also showed an appreciable effect on the removal of NH_3_-N. The effect of voltage on NH_3_-N removal rates was investigated at zeolite dose of 2 g/L, initial NH_3_-N concentration of 30 mg/L and the applied voltage of 9–25 V.

Figure 4 clearly indicates that there was an optimal voltage for the operation, in this work this optimal voltage was 15 V, which corresponded to the maximum removal efficiency of 94% and minimum residue concentration of 1.5 mg/L of NH_3_-N in the solution.

#### Effects of processing time

The effect of zeolite processing time on the removal of NH_3_-N was subsequently investigated by setting the voltage to 15 V with initial NH_3_-N concentration of 30 mg/L and zeolite dosage of 2.0 g/L. The DEP processing time was 30 min. The treated water was sampled and measured every 5 min. As can be seen from Fig. 5, the removal efficiency in the adsorption-DEP process increased considerably with the increase of the processing time. When the processing time went up from 5 to 30 min, the removal efficiency was improved from 70% to 95%, and correspondingly, the residual concentration of NH_3_-N decreased from 9.0 to 1.5 mg/L. The removal efficiency was almost constant after 20 min.

#### Effects of initial concentration

Furthermore, we tested the effect of the initial ammonia concentration vary from 20 to 50 mg/L on the removal efficiency at a fixed zeolite dosage of 2 g/L, voltage of 15 V. It can be seen from Fig. 6 that removal efficiency decreased with the increasing of initial concentration.

### Characterization of the zeolite particles

**Figure 7:**
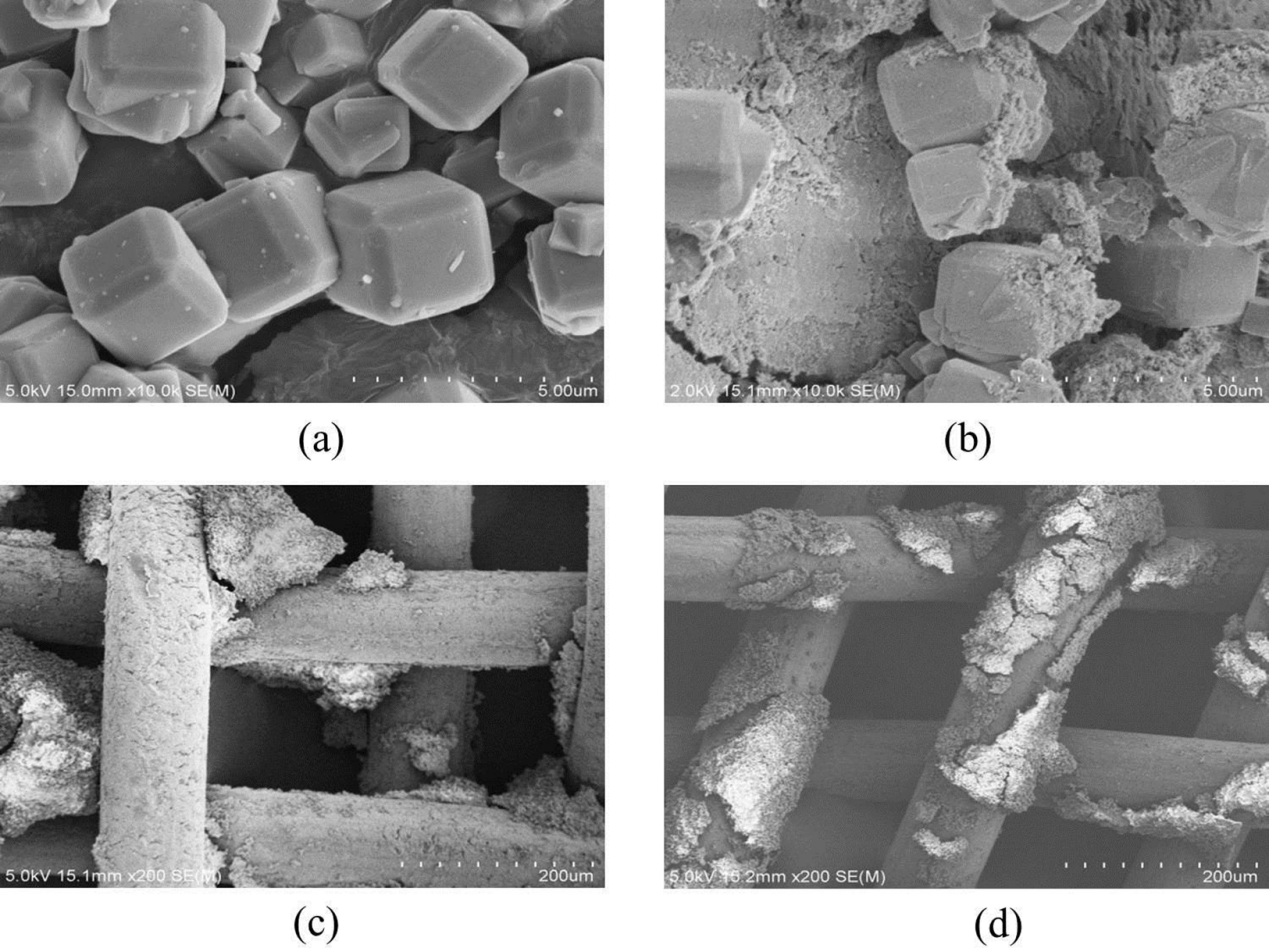
SEM images of the zeolite and electrodes after ADS-DEP process. (A) zeolite after adsorption only; (B) zeolite after ADS-DEP process; (C) positive electrode after ADS-DEP process; (D) negative electrode after ADS-DEP process.

To find out how adsorption and adsorption-DEP processes affected the zeolite particles, we performed SEM examination on the samples taken after each process (Fig. 7).

Scanning electron microscopy image taken from the sample after an adsorption-only process did not show any morphological change in the particles (Fig. 7A). However, the particles were broken down by the adsorption-DEP process, which indicates the important effect of DEP on the particles (Fig. 7B). We have also examined the electrodes after the adsorption-DEP by SEM, and the result is shown in Figs. 7C and 7D. It can be seen that both positive and negative electrodes were deposited by zeolite particles.

## Discussion

### Theory of DEP

**Figure 8:**
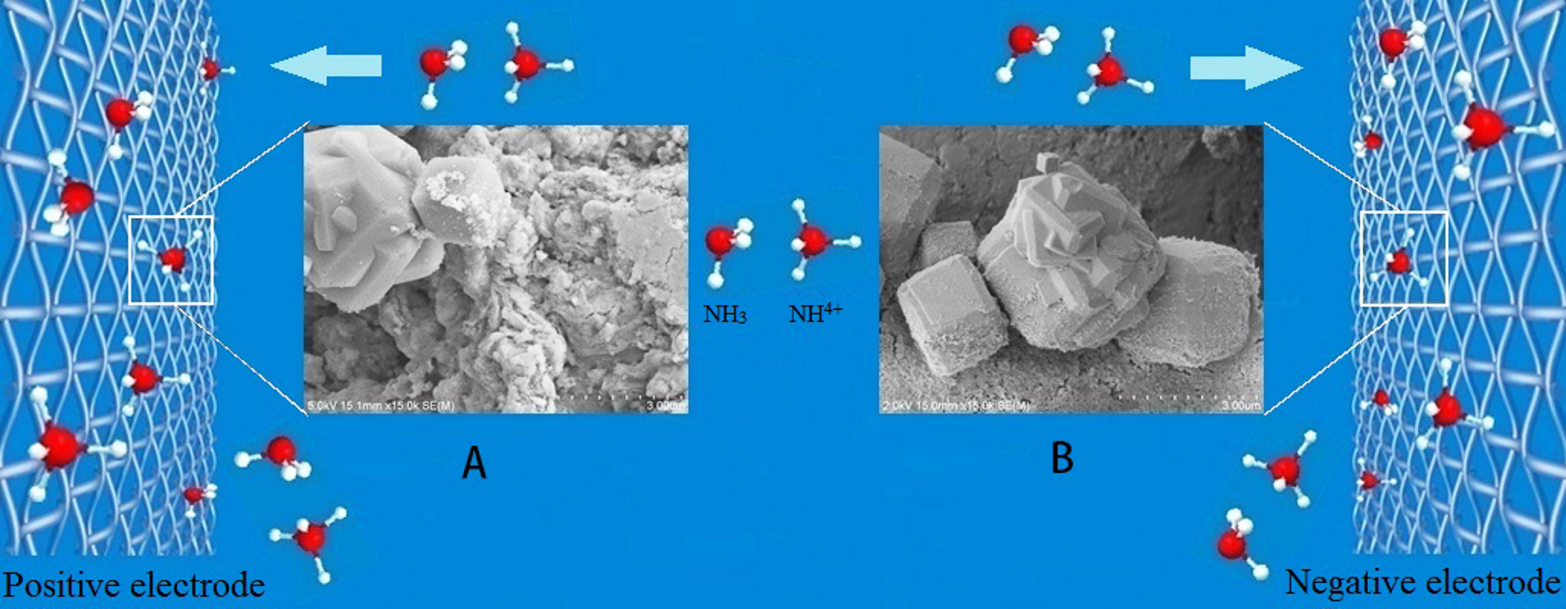
Schematic show of the movement of zeolite particles in NH_3_-N solution towards the electrodes. The voltage used in the DEP was 15 V. (A) SEM image of the zeolite particles trapped on positive electrode; (B) SEM image of the zeolite particles trapped on negative electrode.

We would like to propose the following mechanism to explain the DEP process: a non-uniform electric field generated in the DEP device polarizes adsorbents and induces a dipole moment on each of them. As a consequence, the electric field exerts an unbalanced force on the adsorbent particles, which drives them to move along the electric field gradient in the solution. Those particles near the electrodes were first captured and trapped by the electrodes. Due to the strong electric field in the cross-wire areas of the mesh electrodes, coupled with a possible polarization induction effect between adjacent adsorbent particles, other particles close to the wire junctions were polarized and subsequently trapped by the electrodes. In this way, continuous capture of the adsorbent particles would occur, so more and more adsorbent particles would be trapped and deposited on the electrodes (see Fig. 8). This mechanism is supported by the working principle of DEP—a technique that has been used to manipulate polarized particles suspended in fluid media in non-uniform electric field (Khashei et al., 2015). In the case of a spherical particle, the DEP force *F*_DEP_ is given by the equation below:

(1) $${F_{{\rm{DEP}}}} = 2{\rm{\pi }}{R^3}{{\rm{\varepsilon }}_{\it{m}}}{\mathop{\rm Re}\nolimits} \left[ {K\left({\rm{\omega }} \right)} \right]\nabla {E^2}$$

Where the real part of Clausius–Mossotti factor, Re[*K*(ω)], is defined as:

(2) $$K\left({\rm{\omega }} \right) = {{{\rm{\varepsilon }}_p^*-{\rm{\varepsilon }}_m^*} \over {{\rm{\varepsilon }}_p^* + 2{\rm{\varepsilon }}_m^*}}$$

Where *R* denotes the radius of the particle, ∇*E* the magnitude of the electric field gradient, ε_p_^*^ the complex permittivity of the particle, and ε_m_^*^ that of the media. A non-uniform electric field is necessary to induce a DEP force as stated in Eq. (1) (otherwise ∇*E* = 0). Positive values of Re[*K*(ω)] denote the induction of a positive DEP force that causes particles to be trapped in the regions of high electric field gradient. Negative values of Re[*K*(ω)] denote negative DEP, which means the particles would move towards the regions of low or no electric field.

### Effects of processing factors on the removal efficiency

As can be seen from Fig. 2 (case 1), the adsorption by zeolite was better than bentonite. The NH_3_-N adsorption mechanism by zeolite is mainly chemical adsorption and ion exchange. With the increase of NH_3_-N concentration, more NH_3_-N is available for exchange, which enhance the capacity of exchange reactions (Zhang et al., 2003).

During the DEP process, it was observed that the removal efficiency was largely enhanced when titanium mesh electrodes were employed in the DEP, resulting in much better efficiency (cases 2 and 3 in Fig. 2). Besides, the stainless steel mesh could be electrolyzed slightly during the DEP process. This clearly indicates that DEP can significantly help improve the removal of NH_3_-N as long as a suitable and durable material is used for the electrodes. Based on this initial test result we subsequently chose zeolite as the adsorbent, and titanium mesh as the electrodes, with which we could determine the best possible working conditions for the system.

A phenomenon that the removal efficiency of NH_3_-N increased continuously in an adsorption process only with the increase of the zeolite dosage (Fig. 3) may be straightly understood from the increased adsorption sites of zeolite particles in the system (Wan et al., 2017). We also supposed that two factors caused the optimal zeolite dosage during DEP process in Fig. 3. When in the absence of DEP, the removal rate improved with the increase of zeolite dose, which increase adsorption sites. During the DEP-enhanced adsorption, the adsorbed ion number per unit mass of zeolite could reach highest at the dose of 2 g/L which makes the highest efficiency of DEP. And after the dose of 2 g/L, more adsorbents may weaken polarization ability by DEP process. So any further increase of the amount of adsorbent would lead to a reduced removal efficiency.

It can be seen from Fig. 4 that in the lower voltage region (≤15 V), the removal efficiency was increased with the increase of the voltage. As described in Eq. (1), with the increase of applied voltage, the enhance of electric field gradient increased the DEP force. However, after the voltage reached a certain point, the removal efficiency decreased. This can be explained by DEP principle. Different kinds of particles have their own characteristic voltages, in which the positive DEP could happened strongly. Over the characteristic voltage, the particle would leave the original trapped region (Chen et al., 2009; Lapizco-Encinas et al., 2005). In this work the positive DEP would happen when the voltage was 15 V. This speculation is also consistent with the phenomenon observed by Kim et al. (2004) in a diamond suspension.

Because DEP force was short-distance force, the largely enhanced removal efficiency was due to the fact that with the increase of the processing time, more and more zeolite particles could have chances to be carried to the electrodes by flowing water and trapped on the electrodes, and removed from the solution. By calculating the flowing rate and the device size, the wastewater would flow through all the electrodes and be treated in 20 min. So the removal efficiency of NH_3_-N almost reached the highest in 20 min (Fig. 5). It is also worthy to note that in comparison with a similar adsorption-only result (Wang, Wang & Zhou, 2012), where it took about 1,440 min to reach the adsorption equilibrium of NH_3_-N by zeolite, our adsorption-DEP approach took only 30 min to reach the equilibrium, thus a much faster removal speed. In addition, our approach achieved a significantly higher removal efficiency compared with the adsorption-only method.

It is seen from Fig. 6 that the removal process by adsorption-DEP was particularly powerful at lower initial concentrations: the lower the concentration, the higher the removal efficiency. Figure 6 also shows the difference of the removal efficiency between adsorption-only process and adsorption-DEP process under identical experimental conditions.

### Analysis of zeolite particles by SEM

As seen from Figs. 7A and 7B, the broken-down of the zeolite particles resulted in a significant increase of the particle surface area, thus the number of active adsorption sites to NH_3_-N. This may explain why the DEP-assisted process had significantly enhanced the removal of NH_3_-N in comparison with the adsorption-only method.

Figures 7C and 7D could be understood from the mechanism of DEP rather than electrophoresis. Most of the adsorbent particles were trapped on the cross-wire areas of electrodes where the electric filed gradient was the strongest, indicating that positive DEP took place in this work. Zeolite particles first trapped onto the electrode were polarized again by the electrical field. They produced secondary non-uniform electric field. More particles nearby can be attracted to them because of particle–particle interactions (Ai & Qian, 2010; Cui et al., 2015; Doh & Cho, 2005; Hossan et al., 2013; Jones, 2003), which is illustrated schematically by Fig. 8. Therefore, the NH_3_-N removal efficiency could be improved.

## Conclusion

A new approach for the removal of NH_3_-N in wastewater has been developed in this work using DEP to greatly enhance traditional adsorption method. The factors that affect the removal efficiency have been investigated, and the optimal working parameters determined. The experimental results show that our DEP-enhanced method can significantly improve the removal efficiency of NH_3_-N, in comparison with adsorption-only method, when titanium metal mesh is used as the electrode material and zeolite as the adsorbent. Under identical experimental conditions, this removal efficiency has been found to increase from 66.7% to 95.0%. In addition, the dosage of the adsorbent/zeolite and the processing time have been greatly reduced with the assistance from DEP. Furthermore, we find that a low discharge concentration of NH_3_-N in wastewater, which is as low as 1.5 mg/L after adsorption-DEP treatment, can be achieved, and this is well below the discharge criterion of [NH_4_^+^] < 8 mg/L (the emission standard of pollutants for rare earth industry in China). All these suggest that our adsorption-DEP method provides a high-efficiency approach for the removal of NH_3_-N from wastewater and the technology holds a high potential for future industrial applications.

## Supplemental Information

10.7717/peerj.5001/supp-1 Supplemental Information 1 Raw data.

## Additional Information and Declarations

### Data Availability

The following information was supplied regarding data availability: The raw data are provided in the Supplemental File.
