# Supplementary material for: Highly efficient removal of ammonia nitrogen from wastewater by dielectrophoresis-enhanced adsorption
Source: PeerJ. 2018 Jun 15;6:e5001. doi: 10.7717/peerj.5001 (PMC6005170; doi:10.7717/peerj.5001)
Supplement: Supplemental Information 1 [file peerj-06-5001-s001.docx]

Effect of absorbents and electrode materials

|  | Adsorption (zeolite) | Adsorption (zeolite)/ DEP (Stainless steel) | Adsorption (zeolite)/ DEP (titanium) | Adsorption (bentonite) | Adsorption (bentonite)/ DEP (Stainless steel) | Adsorption (bentonite)/ DEP (titanium) |
| --- | --- | --- | --- | --- | --- | --- |
| Residual (mg/L) | 10.01  9.97  10.06 | 9.28  9.16  8.93 | 4.12  3.87  4.01 | 23.03  22.97  23.01 | 22.26  23.03  22.97 | 18.86  18.95  19.05 |

Effect of absorbent dosage

|  | Adsorption | Adsorption/ DEP |
| --- | --- | --- |
| Residual (mg/L) | 10.72; 10.51; 10.23 | 9.90; 10.05; 10.03 |
|  | 10.22; 10.49; 10.04 | 9.18; 9.35; 9.17 |
|  | 10.24; 9.89; 9.88 | 4.11; 4.00; 4.02 |
|  | 9.03; 9.01; 9.18 | 7.75; 7.89; 7.81 |
|  | 8.00; 8.00; 8.05 | 7.00; 7.12; 7.01 |
|  | 7.68; 7.51; 7.34 | 6.69; 6.50; 6.44 |
|  | 7.13; 6.98; 7.01 | 6.02; 5.91; 6.18 |

Effect of the voltage

| Votage (V) | 9 | 11 | 13 | 15 | 20 | 25 |
| --- | --- | --- | --- | --- | --- | --- |
| Residual (mg/L) | 7.50  7.31  7.62 | 4.03  3.98  4.12 | 3.60  3.69  3.55 | 1.88  1.81  1.71 | 3.53  3.52  3.45 | 2.98  3.05  2.99 |

Effect of processing time

| Time (min) | 5 | 10 | 15 | 20 | 25 | 30 |
| --- | --- | --- | --- | --- | --- | --- |
| Residual (mg/L) | 8.83  8.89  8.71 | 5.32  5.26  5.11 | 3.14  3.01  2.93 | 1.80  1.76  1.91 | 1.50  1.65  1.59 | 1.44  1.50  1.61 |

Effect of the initial concentration (adsorption only)

| initial concentration (mg/L) | 20 | 25 | 30 | 35 | 40 | 45 | 50 |
| --- | --- | --- | --- | --- | --- | --- | --- |
| Residual (mg/L) | 6.31  6.50  6.38 | 7.47  7.61  7.60 | 9.88  9.72  9.92 | 12.11  12.23  11.93 | 14.23  14.06  14.16 | 15.32  14.96  14.72 | 15.79  15.61  15.82 |

Effect of the initial concentration (adsorption/ DEP)

| initial concentration (mg/L) | 20 | 25 | 30 | 35 | 40 | 45 | 50 |
| --- | --- | --- | --- | --- | --- | --- | --- |
| Residual (mg/L) | 0.65  0.49  0.41 | 1.50  1.42  1.40 | 1.84  1.96  1.71 | 3.69  3.51  3.44 | 4.53  4.41  4.59 | 8.24  8.23  8.23 | 12.02  12.06  12.00 |
